# Supplementary material for: The MYC-Regulated RNA-Binding Proteins hnRNPC and LARP1 Are Drivers of Multiple Myeloma Cell Growth and Disease Progression and Negatively Predict Patient Survival
Source: Cancers (Basel). 2023 Nov 21;15(23):5508. doi: 10.3390/cancers15235508 (PMC10705803; doi:10.3390/cancers15235508)
Supplement: Supplementary file 1 [file cancers-15-05508-s001.zip › Seibert et al - Supplementary materials.pdf]

cancers

MDPI

Article

The MYC-regulated RNA binding proteins hnRNPC and LARP1 are drivers of multiple myeloma cell growth and disease progression and negatively predict patient survival

Marcel Seibert [1,2], Sebastian E. Koschade [1,2,3,4], Verena Stolp [1,3], Björn Häupl [1,2,3], Frank Wempe [1], Hubert Serve [1,2,3,4], Nina Kurrle [1,2,3,*], Frank Schnütgen [1,2,3,*], and Ivana von Metzler [1,3,4,*]

1 Goethe University Frankfurt, University Hospital, Department of Medicine, Hematology/Oncology, Frankfurt/Main 60590, Germany
2 German Cancer Consortium (DKTK), Partner Site Frankfurt/Mainz, and German Cancer Research Center (DKFZ), Heidelberg 69120, Germany
3 Frankfurt Cancer Institute, Goethe-University Frankfurt, Frankfurt/Main 60596, Germany
4 University Cancer Center Frankfurt (UCT), University Hospital Frankfurt, Goethe University, Frankfurt am Main, Germany.
* authors contributed equally as supervising authors
Corresponding authors: F. S., schnuetgen@em.uni-frankfurt.de, Tel.: +49-69-6301 4941 and I. v. M., ivana.metzler@kgu.de, Tel: +49-69-6301 6566

Citation: Seibert, M.; Koschade, S.E.; Stolp, V.; Häupl, B.; Wempe, F.; Serve, H.; Kurrle, N.; Schnütgen, F.; von Metzler, I. The MYC-Regulated RNA-Binding Proteins hnRNPC and LARP1 Are Drivers of Multiple Myeloma Cell Growth and Disease Progression and Negatively Predict Patient Survival. *Cancers* **2023**, *15*, 5508. https://doi.org/10.3390/cancers15235508

Received: 5 October 2023
Revised: 2 November 2023
Accepted: 15 November 2023
Published: 21 November 2023

Copyright: © 2023 by the authors. Submitted for possible open access publication under the terms and conditions of the Creative Commons Attribution (CC BY) license (https://creativecommons.org/licenses/by/4.0/).

Supplementary materials:

Data Availability Statement:

The mass spectrometry proteomics data have been deposited to the MassIVE database with dataset identifier MSV000092169 (https://massive.ucsd.edu)." MS data as MaxQuant output, analyzed by Perseus software (v1.6.0.7) are provided in a separated Excel file.

Supplementary Methods

Pathway analysis of proteins obtained from ORA: Enriched proteins, which were differentially regulated in response to MYC depletion, were grouped into REACTOME pathways. Identified pathways were visualized in networks using the EnrichmentMap Cytoscape App [1,2]. Enrichments maps show significantly enriched proteins which were regulated upon MYC knockout and organize them in a network. The nodes of the network represent enriched protein sets involved in REACTOME pathways, for which the node size represents the number of identified proteins being involved in these pathways. The nodes are colored by the adjusted enrichment p-values, which was calculated by g:Profiler, and represents the probability of observing the certain protein enrichment, under the null hypothesis that the observed enrichment is due to random chance. A smaller p-value indicates stronger evidence against the null hypothesis, suggesting that the enrichment is likely to be due to MYC knockout. Edges represent overlaps, since proteins can be involved in different pathways. Many overlaps are expressed as clusters.

Supplementary Results and Discussion

A. SILAC and Mass spectrometry measurements

The three MM-derived cell lines RPMI8226, LP1, OPM2, as well as the stromal control cell line HS5 were transferred in SILAC medium 2 weeks before transduction with either non-target-control (NTC) sgRNA as control or MYC-targeting sgRNA(1) and sgRNA(2) (**Figure S1**). Western blot analysis of the cells used for mass spectrometry analysis revealed that MYC expression was reduced to 25.4% (by sgRNA(1)) and to 11.5% (by sgRNA(2)) in RPMI8226 cells, to 53.6% and 35.3% in LP1 cells, to 20.0% and 20.1% in OPM2 cells and to 32.4% and 29.6% in HS5 cells, respectively (**Figure S1B**).

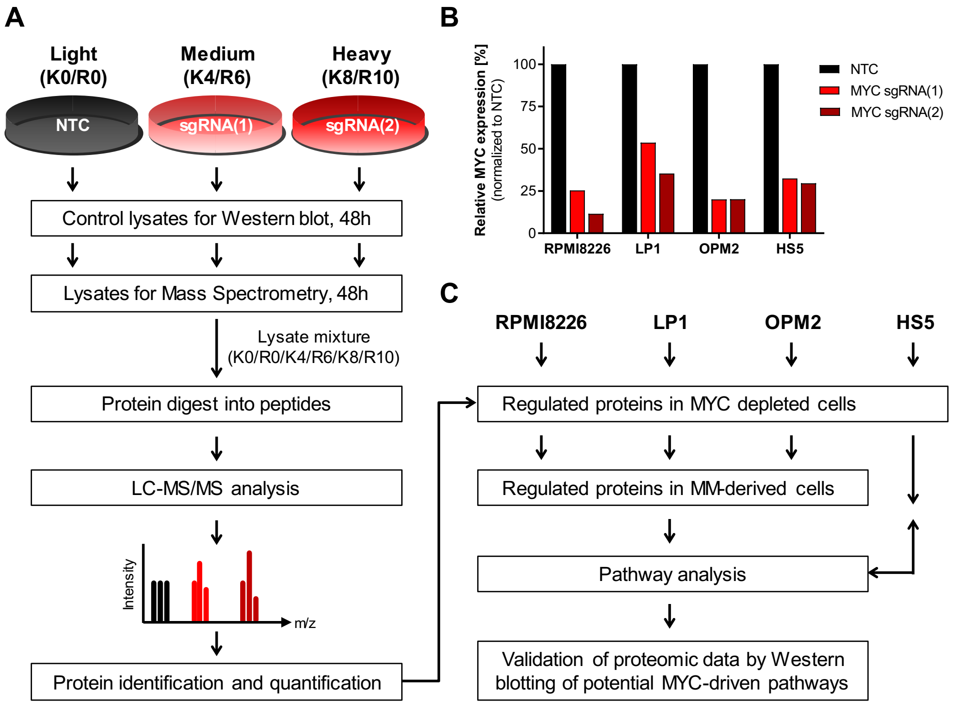

Figure S1 – Schematic SILAC/ proteomic approach. A – Isotopic-labeled cell lysates from three (MM)-derived cell lines and HS5 control cells were prepared 48 hours after transduction with two different MYC-targeting sgRNA. The respective lysates were mixed with NTC in a ratio of 1:1:1 and further processed for mass spectrometry analysis as indicated. Control lysates were prepared for Western blotting, verifying MYC knockout efficiencies. **B** – Quantification of MYC expression determined by Western blotting and assessed two days after transduction. Values were normalized to respective NTC. **C** – Schematic approach for data analysis. HS5 cell-specific regulated proteins were excluded to determine regulated proteins only in MM-derived cell lines, followed by enrichment/ pathway analysis. The identified pathways were analyzed and compared to those determined in HS5 controls.

As shown in volcano plots (**Figure S2**), proteomic changes upon depletion of MYC (compared to NTC) were identified in all cell lines, expressed as mean log_2 fold change (FC) values. All proteins, that were measured as down regulated and exceeded or fell below a previously log_2 (FC) value, are marked in red. Quantified proteins, which were further analyzed based on the enrichment analysis (ORA), such as Eukaryotic Translation Initiation Factor 4E-Binding Protein 1 (4EBP1), La-Ribonucleoprotein 1 (LARP1), Heterogeneous Nuclear Ribonucleoprotein C (hnRNPC), as well as the two known and well-established MYC targets Hexokinase 2 (HK2) and Y-Box Transcription Factor (YB-1) are depicted in the respective volcano plot.

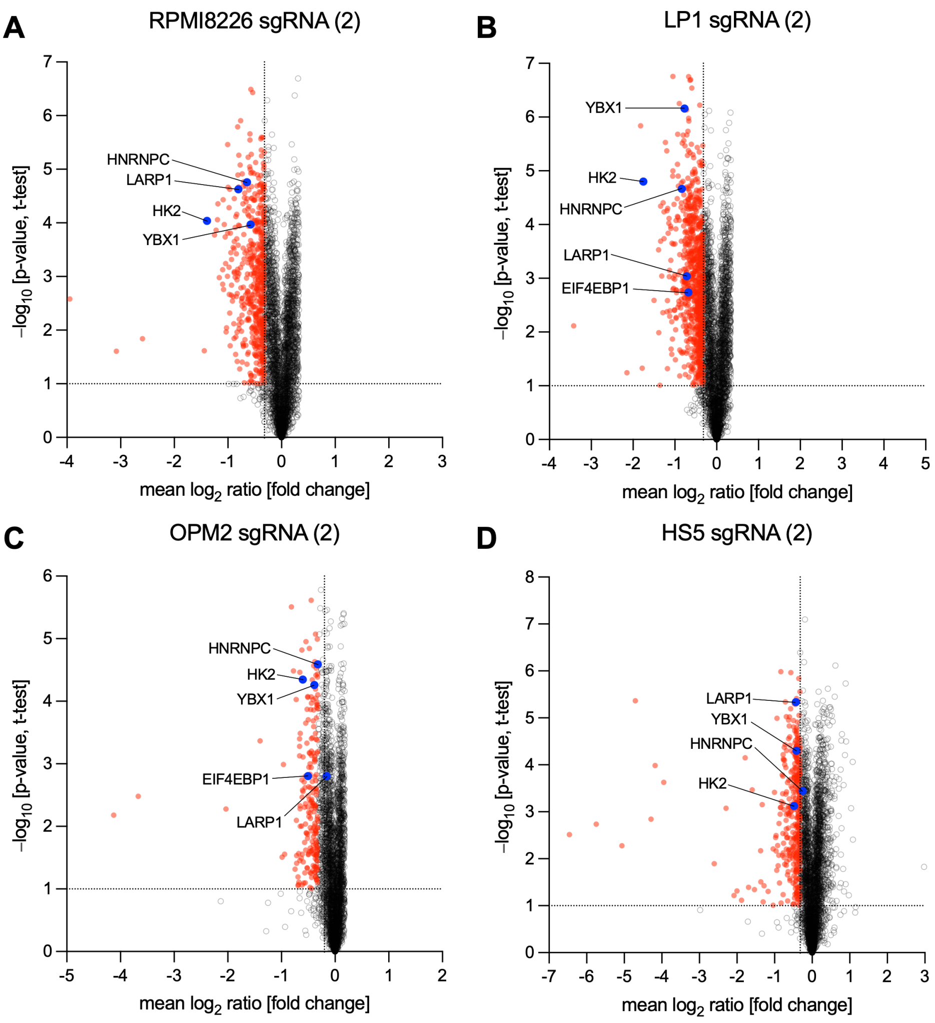

Figure S2 – Volcano plots showing fold changes of protein expression upon MYC depletion. A-D – Log2[fold change] values and the respective p-values (–log10 p-value, t-test, 4 technical replicates) of quantified proteins two days after transduction by MYC targeting sgRNA(2) in cell lines RPMI8226, LP1, OPM2 and HS5 cells as indicated. Red marked proteins were significantly down regulated. The thresholds for significant regulations (log2 [fold change]) were set according to publication-known MYC target proteins (described in [25]). Outlier analysis was per-formed using Benjamini-Hochberg method with false discovery rate (FDR) < 0.05. Further analyzed and literature-known MYC target proteins are labeled in blue.

B. *Quantified MYC-target proteins and data validation*

To assess our proteomic data, literature-known MYC targets were searched for representative proteins [3]. **Figure S3** shows the measured log2 [FC] values of selected known MYC target proteins of the four cell lines, determined upon transduction with MYC targeting sgRNA(1) (left panel) and sgRNA(2) (right panel).

A

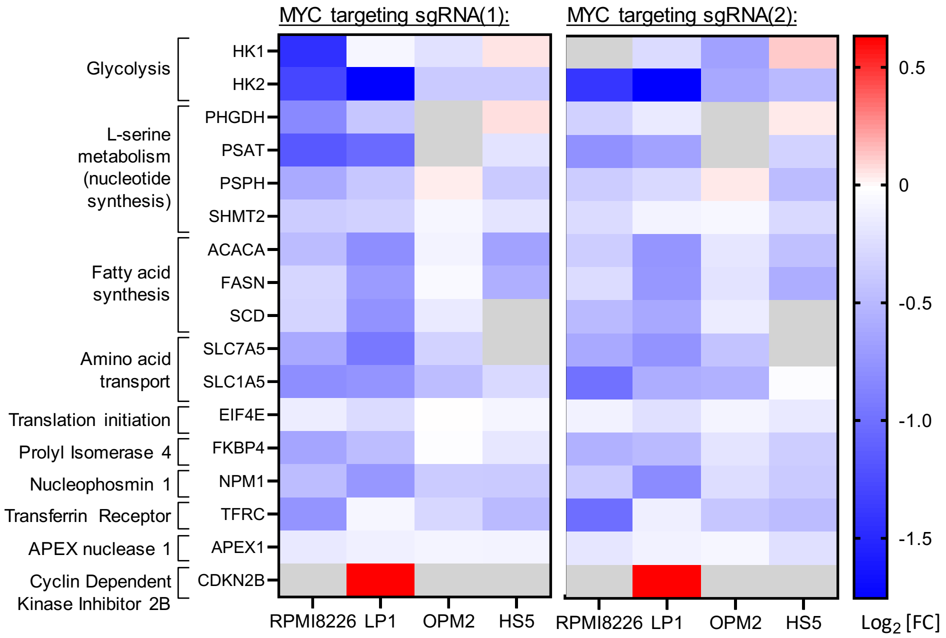

B

RPMI8226	4EBP1	HK2	LARP1	hnRNPC
$\log_2$ [FC]	N/A	-1.27 ±0.06	-0.69 ±0.06	-0.52 ±0.06
$-\log_{10}$ [p]	N/A	4.165 ±0.06	4.36 ±0.14	4.52 ±0.12

LP1	4EBP1	HK2	LARP1	hnRNPC
$\log_2$ [FC]	-0.72 ±0.02	-1.67 ±0.05	-0.74 ±0.01	-0.78 ±0.03
$-\log_{10}$ [p]	3.25 ±0.26	4.36 ±0.22	3.74 ±0.35	5.34 ±0.34

OPM2	4EBP1	HK2	LARP1	hnRNPC
$\log_2$ [FC]	-0.38 ±0.07	-0.47 ±0.07	-0.19 ±0.01	-0.24 ±0.04
$-\log_{10}$ [p]	2.05 ±0.38	4.33 ±0.01	3.29 ±0.24	3.62 ±0.49

HS5	4EBP1	HK2	LARP1	hnRNPC
$\log_2$ [FC]	N/A	-0.41 ±0.04	-0.42 ±0.01	-0.26 ±0.00
$-\log_{10}$ [p]	N/A	3.49 ±0.18	4.52 ±0.41	3.61 ±0.08

Figure S3 – Quantified protein regulations of literature-known MYC targets. A – Heatmap showing protein regulations in RPMI8226, LP1, OPM2 and HS5. Proteins of published MYC-driven pathways were selected and their regulations are shown as $\log_2$ [FC] values. Two different MYC targeting sgRNAs were used (left and right panel, as indicated). Grey marks symbolize proteins which were not identified by mass spectrometry. **B** – Mean $\log_2$ (FC) values and the significance ($-\log_{10}$ p-value) of selected candidate proteins are summarized as means of the regulations by sgRNA(1) and (2) ± SD.

Proteins associated with glycolysis (Hexokinase 1/2 (HK1/2) were identified with negative $\log_2$ [FC] values, confirming these protein as down-regulated upon MYC depletion. In addition, proteins involved in L-serine synthesis, fatty acid synthesis or amino acids transport as well as further known MYC target proteins, like eukaryotic initiation factor 4E (eIF4E), prolyl isomerase 4 (FKBP4), nucleophosmin (NPM1), the transferrin receptor (TFRC) or APEX nuclease (APEX1) were all determined as down-regulated upon MYC depletion, confirming these proteins as MYC target proteins [3] and validating our experimental approach. In contrast, cyclin dependent kinase inhibitor 2B (CDKN2B) was measured as up-regulated (positive $\log_2$ [FC] value) upon MYC knockout in LP1 cells, confirming its established role in repressing MYC expression [3]. CDKN2B was not quantified in the remaining cell lines (indicated by grey boxes in the heatmap). Altogether, the measured $\log_2$ [FC] values for protein regulations upon MYC knockout revealed that mass spectrometry data are in line with published data [3], indicating that our data were independently reproducible and suitable to further identify MYC target proteins.

C. *Exploratory data analysis*

Upon MYC depletion, ORA of proteins regulated in MM-derived cell lines revealed a down-regulation of proteins associated with RNA polymerase 2-mediated transcription

as well as with translation, shown as cluster in enrichment maps, which were generated by using the EnrichmentMap Cytoscape App [1,2] (**Figure S4A**). The node sizes of the enrichment map indicate the numbers of enriched proteins involved in the distinct pathway. However, based on the lower adjusted enrichment p-values (depicted as the node colors, which represents the probability to identify protein enrichments), we followed up with the set of proteins which are associated with translation. To identify pathways that are regulated by MYC in MM-derived cells, we examined the significance of the protein enrichment comparing the MM- and HS5 control protein group. Specifically, the regulated pathways were ranked based on the significance of their protein enrichment, as determined by the enrichment p-value (-log10(p_{adj})). As shown by Manhattan plots (**Figure S4B**), the pathways with low enrichment p-values determined in the MM-derived protein group were numbered and highlighted, revealing that four pathways were less represented in the HS5 control group.

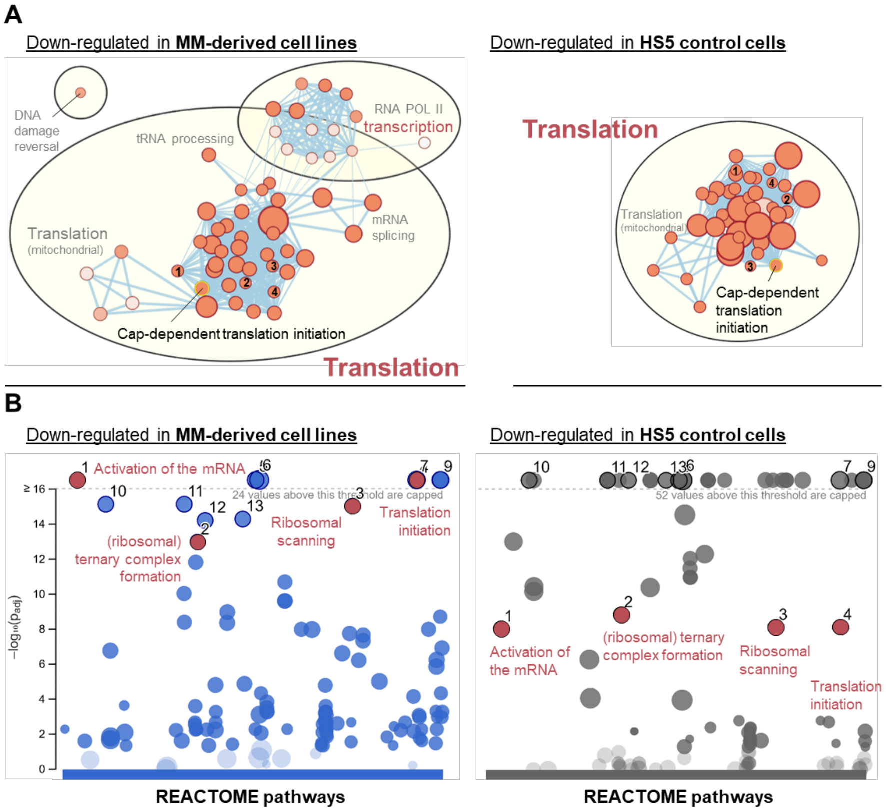

Figure S4 – REACTOME pathways comprising the enriched down-regulated proteins upon MYC depletion. A – Enrichment maps summarizing down-regulated proteins in REACTOME pathways after MYC knockout in MM-derived cell (left panel) and HS5 control cells (right panel). B – Manhattan plots, summarizing the same pathways depicted in A. The y-axis shows the adjusted enrichment p-values in negative log_{10} scale. The –log (p_{adj})-value scale is capped with p-values less than 10E-16. p-values smaller than 10E-16 are all summarized as highly significant. The highest significant pathways, which contains down-regulated proteins enriched in MM-derived cell lines were numbered.

The four determined MYC-regulated pathways (ID number 1-4) were identified as subgroups of the REACTOME pathway annotated as "R-HAS-72737: Cap-dependent Translation Initiation" (**Table S1**). Importantly, a high number of ribosomal subunits (RPL and RPS) in the HS5 control protein group explain the significant enrichment of proteins involved in these subgroups (data not shown).

Table S1 – Enrichment analysis of pathways regulated in MM-derived cells and HS5 controls. The enrichment p-value was calculated by g:Profiler [4]. MM-specific significantly regulated pathways (numbered 1-4) comprise higher numbers of proteins in the MM protein group compared to the HS5 control group.

Most significant affected REACTOME pathways

ID	REACTOME Pathway	p_{adj}-value (MM-derived cell lines)	p_{adj}-value (HS5 control)
**1**	Activation of the mRNA upon binding of the cap-binding complex and eIFs, and subsequent binding to 43S	9.13 E-18	8.92 E-09
**2**	Formation of the ternary complex, and subsequently, the 43S complex	1.01 E-13	1.39 E-09
**3**	Ribosomal scanning and start codon recognition	9.33 E-16	7.30 E-09
**4**	Translation initiation complex formation	8.13 E-17	7.30 E-09
5	Major pathway of rRNA processing in the nucleolus and cytosol	6.47 E-24	7.34 E-76
6	Metabolism of RNA	8.91 E-38	4.38 E-54
7	Translation	2.76 E-18	2.02 E-45
8	rRNA processing in the nucleus and cytosol	4.83 E-23	2.32 E-83
9	rRNA processing	1.19 E-23	3.98 E-84
10	Cap-dependent Translation Initiation	7.66 E-16	4.95 E-38
11	Eukaryotic Translation Initiation	7.66 E-16	4.95 E-38
12	GTP hydrolysis and joining of the 60S ribosomal subunit	6.46 E-15	3.34 E-39
13	L13a-mediated translational silencing of Ceruloplasmin expression	5.33 E-15	2.29 E-39

$-\log_{10}(p_{adj}\text{-value})$: 0 5 10 15 20 25 30 35 40 45 ≤50

Altogether, this finding suggests that while MYC depletion results in a general response of down-regulating translation factors, certain sub-processes are specifically affected in MYC-depleted MM cell lines. The differential regulation of these pathways could have important implications for understanding the molecular mechanisms underlying a more detailed role of MYC in the identified oncogenic translation program described in MM cells [5].

References

1. Shannon, P.; Markiel, A.; Ozier, O.; Baliga, N.S.; Wang, J.T.; Ramage, D.; Amin, N.; Schwikowski, B.; Ideker, T. Cytoscape: a software environment for integrated models of biomolecular interaction networks. *Genome Res.* **2003**, *13*, 2498–2504. doi:10.1101/GR.1239303.
2. Merico, D.; Isserlin, R.; Stueker, O.; Emili, A.; Bader, G.D. Enrichment map: a network-based method for gene-set enrichment visualization and interpretation. *PLoS One* **2010**, *5*. doi:10.1371/JOURNAL.PONE.0013984.
3. Zeller, K.I.; Jegga, A.G.; Aronow, B.J.; O'Donnell, K.A.; Dang, C. V. An integrated database of genes responsive to the Myc oncogenic transcription factor: identification of direct genomic targets. *Genome Biol.* **2003**, *4*, R69. doi:10.1186/GB-2003-4-10-R69.
4. Raudvere, U.; Kolberg, L.; Kuzmin, I.; Arak, T.; Adler, P.; Peterson, H.; Vilo, J. g:Profiler: a web server for functional enrichment analysis and conversions of gene lists (2019 update). *Nucleic Acids Res.* **2019**, *47*, W191–W198. doi:10.1093/NAR/GKZ369.
5. Manier, S.; Huynh, D.; Shen, Y.J.; Zhou, J.; Yusufzai, T.; Salem, K.Z.; Ebright, R.Y.; Shi, J.; Park, J.; Glavey, S. V.; et al. Inhibiting the oncogenic translation program is an effective therapeutic strategy in multiple myeloma. *Sci. Transl. Med.* **2017**, *9*. doi:10.1126/SCITRANSLMED.AAL2668.
